# Death recollection moderates stress-influenced depression in Thai boarding school students

**DOI:** 10.1186/s40359-025-03147-4

**Published:** 2025-07-29

**Authors:** Justin DeMaranville, Tinakon Wongpakaran, Nahathai Wongpakaran, Danny Wedding

**Affiliations:** 1 Mental Health Program, Multidisciplinary and Interdisciplinary School (MIdS), Chiang Mai University, Chiang Mai, 50200 Thailand; 2 Department of Psychiatry, Faculty of Medicine, Chiang Mai University, Chiang Mai, 50200 Thailand; 3 Department of Clinical and Humanistic Psychology, Saybrook University, Pasadena, CA 91103 USA; 4 Department of Psychology, University of Missouri-Saint Louis, St. Louis, MO 63121 USA

**Keywords:** Buddhism, Death meditation, Death contemplation, Stress, Depression, Teenager, Boarding, Meditation

## Abstract

**Background:**

Death recollection is a form of mindfulness meditation that orients a practitioner’s calm attention toward an awareness of death. This meditation is practiced by Theravada Buddhists of all ages throughout Thailand. This research investigates how recollecting death influences Thai teenager mental health.

**Methods:**

Purposive and convenience sampling methods were used to recruit participants from five boarding schools in northern Thailand. Students aged 15–18 were invited to participate, and they completed the questionnaires Perceived Stress Scale (PSS), Outcome Inventory: Depression Subscale (OI: Depression), and Inner Strength Based Inventory: Meditation (iSBI: Meditation). Moderation analysis was conducted with SPSS ver. 27 and PROCESS ver. 4.2.

**Results:**

The sample comprised 440 students (88.2% female) with a mean age of 16.34 ± 0.96. This population had moderate stress (24.08 ± 5.04), low depression (3.82 ± 3.39), and an ‘often but not every day’ average meditation frequency (2.92 ± 1.38). There were 42 students (10.2%) who practiced death recollection in the past month. As hypothesized, death recollection practice moderated the relationship between stress and depression, indicating those who practiced may have had fewer symptoms of depression due to stress. The moderation effect was significant: B = 0.133, standard error = 0.061, 95% CI = .253 to .013 after controlling for the meditation frequency of the population.

**Conclusions:**

The significant moderation effect suggests that death recollection may negatively influence how stress can contribute to the development of depression symptoms in boarding school students. A longitudinal study is recommended to confirm variable interaction across time for assessing death recollection as a causal influence of stress influenced depressive symptoms. This would clarify whether long-term practice strengthens moderation over time.

**Highlights:**

1. The recollection of death is observed even among adolescents.
2. Recollection of death is discovered to mitigate depression resulting from stress.
3. Recollection of death is recommended after calming oneself with concentration meditation.

## Introduction

Death recollection (e.g., maranasati in Buddhism) is a form of mindfulness meditation in which death is contemplated to understand its significance [46]. Recollecting death is often preceded by concentrating the mind with breathing meditation or another samādhi (concentration) meditation. Breathing meditation and breathing exercises have been shown to help with stress reduction [10, 33, 55]. Having calmed oneself, the meditator’s mind can turn toward contemplating mortality. According to Buddhist scriptures, death recollection involves contemplating that death could occur tomorrow, tonight, or even after the very next breadth [43]. The meditator may hold in mind that he or she has only minutes left to live, allowing reactive thoughts and feelings that follow to pass as one’s meditative concentration deepens. How much focus a person gives to death recollection is often individually specific or subject to instruction that a person receives, with different methods for how death is contemplated. That all things arise and pass away impermanently is one of the primary insight that death recollection engenders, with the Buddhist path offered as a means to develop further meditative insight and an ethical lifestyle in light of that knowledge.

Of the ten recollections (topics to meditate upon) listed in the Pali scriptures and practiced in Theravada Buddhism, death recollection comes last. The first seven recollections (anussati) include contemplating the qualities of the Buddha, dhamma, sangha, virtue, dana, devas, and nibbana. The remaining three recollections are of breathing, body, and death, all of which are actually mindfulness (sati) practices, though all retain the label of recollections [3, 46].

Meditation types vary, each with more or less emphasis on attention or contemplation (such as about death), with different benefits associated with different practices [41]. Mindfulness meditation has been documented to be a useful tool in helping adolescents deal with stress, depression, and anxiety, as well as improving self-regulation, particularly in response to stress [2, 13, 39]. A growing body of empirical studies and clinical experience suggest that incorporating mindfulness practices (as found in meditation) will enable clinicians to help youth cope with challenging symptoms more effectively [34, 60].

It is unknown how many meditators practice death recollection in Thailand, though it is generally practiced by Buddhists for religious development [27]. Death recollection may be considered more advanced as it is generally practiced following a concentration meditation (*Samatha*) such as mindfulness of breathing, whereas concentration meditations are not often preceded by other styles. The Visuddhimagga (Buddhist scriptural commentary) highlights how attention should be appropriate before practicing death recollection in order to see impermanence without anxiety, suggesting that cultivating the appropriate state of mind beforehand is beneficial [3]. In Thailand, death meditation is included as a standard practice for monks who teach meditation to community members. These monks typically reside in temples, which serve as centers of spiritual life and community welfare. Thai temples depict death prominently — in murals, on tombs, through the display of skeletons, and as frequent subjects in dhamma talks and chants. Death recollection, like loving-kindness meditation, is discussed across all age groups and is taught in some schools, particularly schools affiliated with a temple. These schools may be partially administered by monks who help guide the spiritual education of the children. In the temple or school, Buddhism has a long history of integrating an understanding of death into Thai people’s conception of the life course. This embrace of death as a part of life may have been relevant as Thai society processed the implications that COVID-19 posed on the lives of everyone.

The COVID-19 pandemic disrupted school schedules and social stability, contributing to widespread psychological distress among adolescents. The global prevalence rates of mental illness during the pandemic varied widely, with stress ranging from 7 to 25% and depression from 6.3 to 71.5% [29]. In contrast, pre-pandemic data of Thai high school students reported that 14% had depression [5]. Excessive stress may have an attritional influence on the development of depression, particularly in disaster contexts [13, 28]. Covid-related stress was found to increase rumination, which is a repetitive cycle of negative thought patterns, leading to higher depression symptoms [40]. Cognitive processes such as rumination are important influences for depression, whereas mindfulness as developed in meditation may be an example of positive cognition capable of countering ruminative thoughts. Trait mindfulness has been found to moderate the influence of Covid-related stress on rumination [40], suggesting the value of positive cognition and meditative awareness. Another study on Thai adolescents found rumination and emotional regulation explained the relationship between mindfulness and psychological distress [17], linking together these cognitive processes with mental health outcomes. Social stressors found to contribute to depression are multifaceted and include acculturative stress, stress related to parents, or being bullied [13, 28].

Biologically, stress can dysregulate the hypothalamic-pituitary-adrenal (HPA) axis and amplify ruminative thought patterns [21, 57]. These maladaptive stress responses disrupt emotional regulation and increase the risk of depression. Interventions that enhance adaptive cognitive reappraisal and emotional regulation may counteract these stress-induced vulnerabilities [21]. An 18-week mindfulness meditation study found significant differences in biological stress markers, including skin conductance and temperature, between meditators and controls, suggesting mindfulness may have measurable physiological stress-reducing effects [33].

In this study, the authors’ objective was to explore how death recollection practice moderated the relationship between stress and depression among high school students. The authors hypothesized that death recollection would buffer the influence of perceived stress on depressive symptoms. This hypothesis follows current theory about the reciprocal interaction between stress and depression [21] and the potential for meditation to reduce the severity of mental illness symptoms [2, 13, 23]. The literature about death recollection and death education [26, 59], in conjunction with the benefits reported about mindfulness meditation in adolescents [39], suggests death recollection may influence mental health and depression outcomes.

## Materials and methods

This study employed a cross-sectional survey. The students were invited to participate in five boarding schools in Northern Thailand between July and August 2021. Two boarding schools were Buddhist, purposively selected to ensure enough meditators were included in the study. These schools were selected due to their focus on Buddhist practices, which align with the research objectives. Students at Buddhist boarding schools were encouraged to practice meditation, unlike those in non-religious schools, which may not offer meditation instruction. Both Buddhist boarding schools agreed to participate in the study after discussions with the research team. The remaining three secular schools were recruited on a first-come, first-served basis in response to emails and phone calls from a research assistant who provided information about the study. Electronic and paper surveys were provided to the students at their schools. Boarding school students were selected due to their relatively cloistered lifestyle, which offers a degree of environmental and social homogeneity. Unlike day students, they share similar routines, social exposures, and institutional structures, reducing extraneous variability from the day-to-day lives of the participants. Inclusion required student status at a northern Thailand boarding school, aged 15–18, and studying in grades 10–12. The sample size for multiple linear regression was determined by an anticipated effect size of 0.15, a significance level (alpha) of 0.05, and a power (beta) of 0.9, while accounting for seven predictors that included sociodemographic information and a meditation control [42]. The calculation indicated that 129 participants were necessary for the analysis. The data originated from a sample of 453 participants collected for structural equation modeling and mediation analysis, with the full sample being used in this secondary study to increase statistical power. Ethical approval for this study was obtained from the Research Ethics Committee, Faculty of Medicine, Chiang Mai University, Thailand (study code 236/2021, approved 10 June 2021).

## Instruments

### Outcome inventory (OI-21) depression subscale

The OI-21 can be reliably used in clinical and non-clinical populations to measure four types of mental distress (depression, anxiety, somatization, and interpersonal difficulties) over the last month. A total score or four subscale scores reflecting each mental health domain can be calculated. There are five items in the OI-depression subscale that measure five symptoms: negative view of life, hopelessness, lack of goals, depression, and suicidality. The items are rated with a five-point Likert range from “never” to “always.” The depression subscale total score ranges from 0 to 20, with seven used as a cutoff for major depression [16, 51, 53]. The Cronbach alpha value was 0.92 in first-year Thai medical students [30]. The Cronbach alpha value in this study was 0.798.

### Perceived stress scale (PSS-10)

The Perceived Stress Scale has worldwide use for the assessment of an individual’s perception of stress over the past month [7]. The tool’s ten items utilize a five-point Likert scale with responses from “never” to “very often.” High scores indicate a greater perception of stress. Six questions assess stress symptoms, and four questions are controls. The total score ranges from 0 to 40, with scores between 14 and 25 considered moderate [49] and higher scores equated as ‘high stress’. The Thai PSS-10 has cross-cultural validity and good internal validity of 0.85 in medical students and clinical patients [52]. The Cronbach’s alpha in this study was 0.70.

### Meditation evaluation questionnaire

Participants were asked about their meditation practices over the last month. Seven common meditation styles were listed: vipassana, manomayiddhi, Buddha image visualization, color kasina, death recollection, Anapanasati (breathing meditation), and mindfulness.

### Inner strength-based inventory: meditation (iSBI)

This ten-item inventory measures the frequency of behaviors emblematic of the Buddhist ten perfections (e.g., loving-kindness, truthfulness, perseverance, generosity, morality, mindfulness, wisdom, patience, endurance, and equanimity). Each strength is measured with a five-point, multiple-choice answer along an ordinal scale. The meditation frequency question used in this study corresponds to the ‘mindfulness’ characteristic in the ten perfections. The person reliability for the iSBI by Rasch analysis is 0.86. The two-week test-retest score for the intraclass coefficient was 0.88 [54].

### Statistical analysis

Descriptive analysis examined demographic data using percentages, means, and standard deviations for variable scores. Continuous variables were analyzed with t-test and ANOVA. Chi-squared test assessed differences between sociodemographic data and variables. Pearson correlation and point biserial correlation were used to assess variable relationships.

To assess the model, assumption testing with linear regression was used. The Tukey test detected 13 outliers which were removed. The dependent variable, depression, was log-transformed to address nonlinearity. The remaining data were normally distributed and homoscedastic. No multicollinearity was demonstrated.

**Fig. 1:**
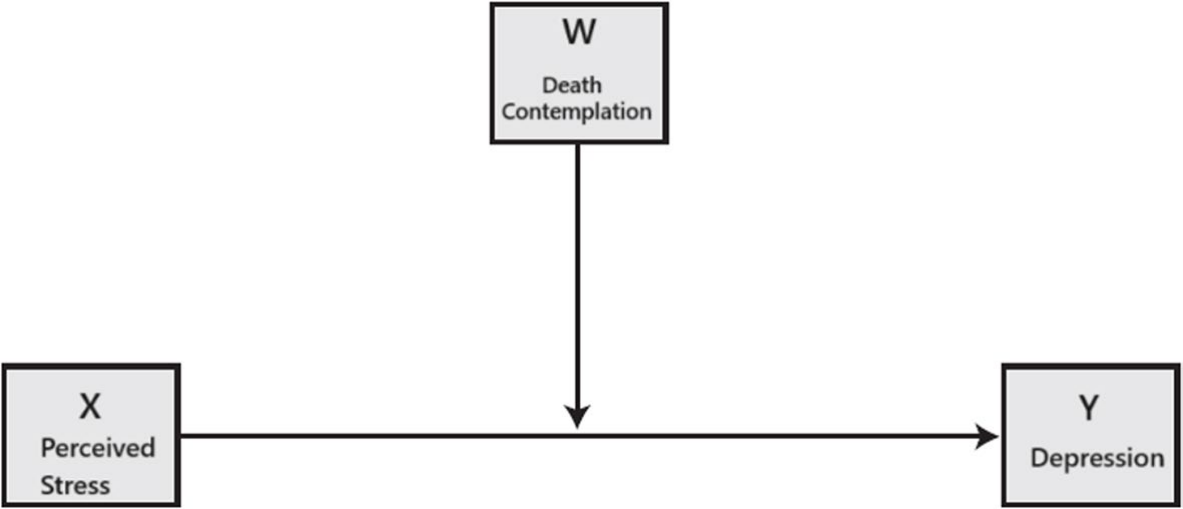
Moderation effect of death contemplation on depression

Multiple regression analysis and moderation analysis were conducted to identify significant predictors for depressive symptom scores. In the moderation models, we adopted Model 1 based on Hayes [15], where perceived stress scores served as the antecedent variable (X), depressive scores as the outcome variable (Y), and death recollection practice (Yes or No) as the moderator (W) (Fig. 1). We scrutinized significant interactions (perceived stress scores by death recollection state) by visualizing predicted values of depressive symptoms within the presence or absence of death recollection. Additionally, significant demographic variables with depression and death recollection were employed as covariates. In conducting moderation analyses, we employed resampling or bootstrapping, following the product of coefficient strategies as suggested by Preacher and Hayes [15, 36]. This process was carried out using PROCESS, Version 4.2, an add-on statistical analysis for SPSS developed by Hayes [16]. Interpretation of results from PROCESS included standard errors, *p*-values, and confidence intervals for the direct effect coefficients and bootstrap confidence intervals. Statistical significance was indicated by confidence intervals that did not straddle zero. The level of significance for all analyses was set at *P* < 0.05. All statistical procedures were performed using IBM SPSS, Version 27.0.

## Results

Descriptive statistics (Table 1) highlight sociodemographic information, covariate distributions, and mental health averages. Most participants were female (88.2%). The number of students from Buddhist (53.6%) and secular schools (46.4%) were nearly equal, though the number of death recollection meditators from Buddhist schools was greater (76.2%). Among all participants, 3.4% experienced low stress, 56.6% experienced moderate stress, and 40% experienced high stress. Additionally, 20.9% of the population met the screening cutoff score for depression. Overall, 83.2% of the participants meditated at least on some occasions, and 38.6% meditated daily at least once. Approximately 11% of the meditators practiced death recollection. The meditators who practiced death recollection had a significantly higher meditation frequency 3.83 ± 1.40 <.001 compared with those who did not practice death recollection 2.83 ± 1.34. The perceived stress of those who practiced death recollection were significantly lower *p* =.010, as were the scores for depression *p* =.042.

**Table 1:** Demographic characteristics of study participants

Variables	N or mean ± SD (%)			Difference (χ^2^ or *t*-test)	*p*-value
	ALL (*n* = 440)	No Death recollection (*n* = 398)	Death recollection (*n* = 42)		
Age (15–18)	16.34 ± 0.96	16.34 ± 0.95	16.31 ± 1.00	*t* (438) =.207	.836
Sex—female	388 (88.2%)	358 (89.9%)	30 (71.4%)	χ^2^(1) = 12.505	.002
Sex—male	52 (11.8%)	40 (10.1%)	12 (28.6%)		
School Type—Buddhist	236 (53.6%)	204 (51.3%)	32 (76.2%)	χ^2^(1) = 9.498	.002
School Type—Secular	204 (46.4%)	194 (48.7%)	10 (23.8%)		
Religion—Buddhism	393 (89.3%)	356 (89.4%)	37 (88.1%)	χ^2^ (1) =.073	.792
Religion—Non-Buddhist	47 (10.7%)	42 (10.6%)	5 (11.9%)		
Income ≤ USD 295*	235 (53.4%)	222 (55.8%)	13 (31%)	χ^2^(1) = 9.410	.003
Income ≥ USD 296	205 (46.6%)	176 (44.2%)	29 (69%)		
Meditation frequency (1–5)	2.92 ± 1.38	2.83 ± 1.34	3.83 ± 1.40	*t* (438) = − 4.602	<.001
rarely or never	74 (16.8%)	69 (17.3%)	5 (11.9%)	χ^2^(4) = 28.993	<.001
some occasions	133 (30.2%)	129 (32.4%)	4 (9.5%)		
often but not every day	63 (14.3%)	61 (15.3%)	2 (4.8%)		
every day, on time	92 (20.9%)	79 (19.8%)	13 (31%)		
every day, multiple times	78 (17.7%)	60 (15.1%)	18 (42.9%)		
Perceived Stress Scale- PSS (0–40)	24.08 ± 5.04	24.33 ± 4.84	21.67 ± 6.21	*t* (46.4) = 2.697	.010
Outcome Inventory: Depression (0–20)	3.82 ± 3.39	3.93 ± 3.39	2.81 ± 3.23	*t* (438) = 2.044	.042

* 1 USD = 32 THB (exchange rate at time of study)

### Correlation

The correlation between perceived stress and depression (*r* =.541, *p* <.01) had a moderate effect size (Table 2). Death recollection was found to have a small, negative relationship with stress (*r* = −.156, *p* <.01) and depression (*r* = −.097, *p* <.05). Regarding covariates, Buddhist schools, as opposed to secular schools, had a small association with death recollection practice (*r* =.146, *p* <.01). Buddhist religion was significantly associated with school type (*r* =.238, *p* <.01), meditation frequency (*r* =.410, *p* <.01). and death recollection (*r* =.147, *p* <.01).

**Table 2:** Correlation matrix among variables

	1	2	3	4	5	6	7	8	9
1. Age	1								
2. Sex (female)	−0.032	1							
3. School Type (Buddhist)	−.160^**^	−.208^**^	1						
4. Religion (Buddhist)	−.119^*^	−.185^**^	.238^**^	1					
5. Family Income (high)	−.116^*^	−0.058	.190^**^	0.047	1				
6. Meditation Frequency	−0.029	−.128^**^	.184^**^	.410^**^	.217^**^	1			
7. Death Recollection (yes)	−0.010	−.169^**^	.146^**^	.147^**^	−0.013	.215^**^	1		
8. Perceived Stress	−0.083	0.062	−.135^**^	−.225^**^	−0.016	−.247^**^	−.156^**^	1	
9. Depression	−0.024	0.064	−.205^**^	−.207^**^	−.112^*^	−.326^**^	−.097^*^	.541^**^	1

*p <.05

** *p* <.01

### Regression

The interaction effect of death recollection on the stress and depression relationship was found to have a small and significant effect (B = − 0.044, *p* = 0.014, (95%CI −.079, −.009), controlling for religion, school type, age, sex, income, and meditation frequency (Table 3). Of the covariates included in the final model, only income (B = − 0.134, *p* <.047) and meditation frequency (B = − 0.123, *p* <.001) were significant. The *r*-squared value was 0.399 in the final model. The interaction suggests that death contemplation moderated the stress and depression relationship.

**Table 3:** Model estimations of depressive symptoms

Model		*B*	SE	*t*	*p*-value	95% Confidence Interval
1	Constant	1.275	0.032	39.330	<.001	(1.211, 1.338)
R^2^ = 0.332	Perceived stress (X)	0.095	0.006	14.756	<.001	(.082,.108)
2	Constant	1.280	0.034	37.740	<.001	(1.214, 1.347)
R^2^ = 0.343	Perceived stress (X)	0.101	0.007	14.425	<.001	(.087,.115)
	Death recollection (W)	−0.173	0.117	−1.477	.140	(−.403,.057)
	Interaction (XW)	−0.047	0.018	−2.555	.011	(−.083, −.011)
3	Constant	2.152	0.578	3.724	.0002	1.016, 3.288)
R^2^ = 0.399	Perceived stress (X)	0.091	0.007	12.922	<.001	(.077,.105)
	Death recollection (W)	−0.043	0.117	−0.365	.715	(−.272,.187)
	Interaction (XW)	−0.044	0.018	−2.456	.014	(−.079, −.009)
	Age	−0.022	0.034	−0.648	.517	(−.089,.045)
	Sex	0.072	0.101	0.714	.476	(−.126,.269)
	Religion	−0.103	.106	−0.971	.332	(−.310,.105)
	School type	−0.037	0.071	−0.516	.606	(−.177,.103)
	Income	−0.134	0.067	−1.996	.047	(−.267, −.002)
	Meditation frequency	−0.123	0.026	−4.700	<.001	(−.175, −.072)

*B* Unstandardized coefficients, *SE* Standard error

**Fig. 2:**
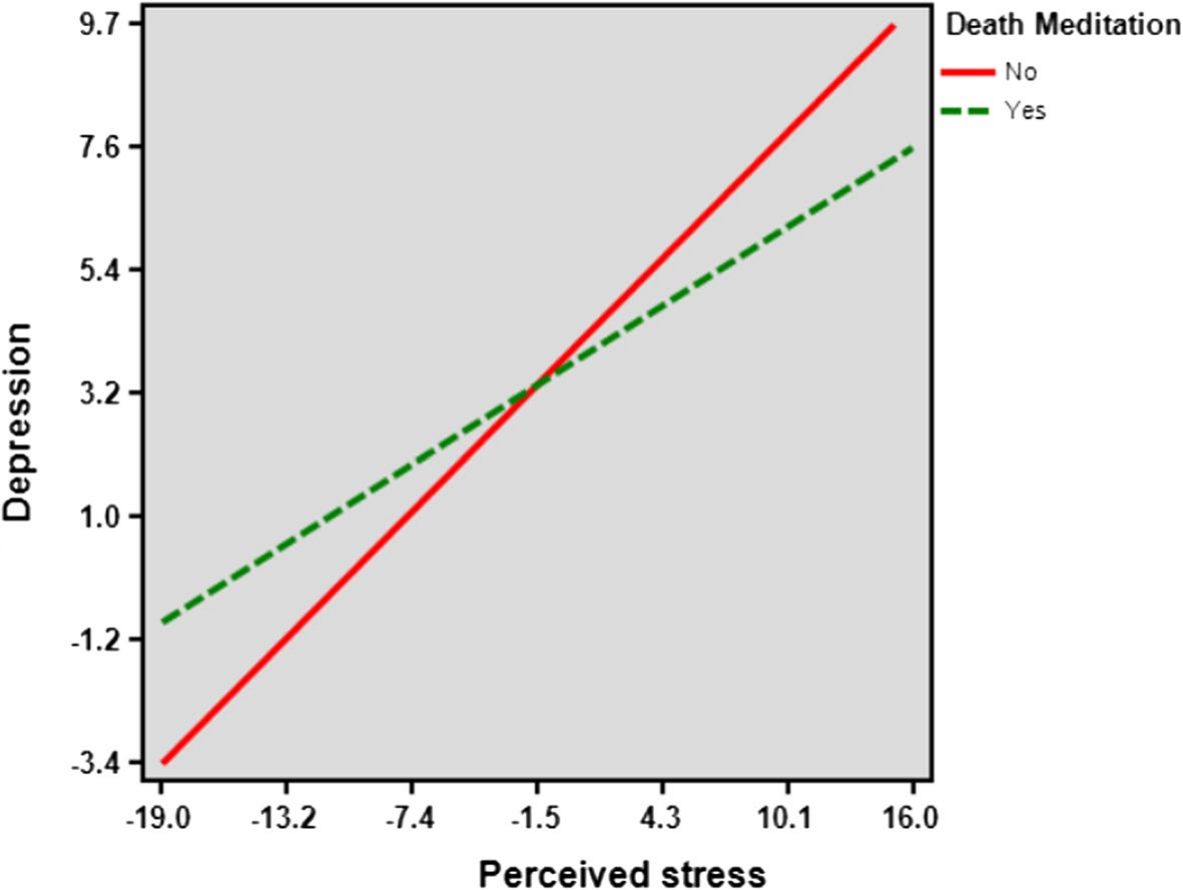
Regression lines between depression and perceived stress scores based on the level of death recollection

In absence of death recollection, the slope coefficient (*B*) was 0.384, t(449) = 13.22, *p* <.001, whereas in the presence of death recollection, the slope coefficient (*B*) was) found to be significant 0.241, t(449) = 3.80, *p* <.001, indicating less perceived stress influence on depressive symptoms (Fig. 2). A significant difference between the two slopes was noted.

## Discussion

This study’s primary hypothesis, to discern the moderating influence of death recollection on the relationship between stress and depression, was found to be significant. It may be that death recollection buffered the effects of stress, resulting in fewer depressive symptoms in this population. However, these findings are not sufficient to confirm that theory. This population of teenage boarding school students in northern Thailand is unique. Though their depression scores were low, a minority of the participants did meet the screening cutoff for depression. This may be due to the high proportion of teenage depression found during the COVID-19 period [29]. Income was found to be the only significant sociodemographic variable in the moderation model. The negative association between higher income and depression outcomes is in line with other research that highlighted how lower-income households experience higher stress, sadness, and suicidal ideation [6]. While this research on death meditators found that contemplating death may result in fewer depressive symptoms, it must be acknowledged that other important factors are at play. Therefore, researchers should further explore the scientific evidence and potential clinical applications of these findings.

Building on this, the relationship between stress and depression is well-documented, with chronic stress often exacerbating depressive symptoms. However, emerging research suggests that this relationship may differ across individuals—particularly among meditation practitioners, whose cognitive and emotional responses to stressors may shift as a result of contemplative training. One key factor within this dynamic is death recollection, a structured meditative practice involving deliberate reflection on mortality. Unlike intrusive or anxiety-driven thoughts about death, formal death recollection is often practiced as part of an advanced meditation practice. Rather than acting as a passive intermediary of stress (i.e., mediator), we propose that it moderates the stress-depression link by altering how practitioners appraise and respond to stressors.

We ground this interpretation in two key theoretical frameworks: the stress-appraisal-coping model [20] and terror management theory (TMT), adapted with a contemplative lens. Within the stress-coping model, outcomes hinge on appraisals—whether stressors are seen as threats, challenges, or neutral. Death recollection may reframe stress appraisals by reinforcing impermanence (e.g. “This too shall pass”), which may lessen the threat surrounding perceived stress. Stressors related to the COVID-19 pandemic, such as fears about illness or death, were unique in light of the portrayal of the disease in the media. The COVID-19 pandemic had, according to Fairlamb, increased death-related information and heightened the public’s awareness of death, and those existential concerns may have influenced the development of depression [9]. A meditator who practiced death recollection may have found relief in viewing death with impermanence, reducing the influence that pandemic related information may have had. Classic TMT posits that mortality awareness induces anxiety and self-preservation drives. However, meditative death recollection may reduce this anxiety by nurturing acceptance (e.g., “I am not afraid because I understand impermanence”), shifting the response away from ego defense and toward equanimity [24]. In our sample, we hypothesized that death recollection would weaken the association between stress and depression, and this may have been accomplished by stressors having been reframed through impermanence, by reducing catastrophizing, and through normalizing emotional responses. For example, a stressed meditator recalls, “All things fade—this difficulty is temporary.” Additionally, emotional regulation may be enhanced as death recollection normalizes mortality, which may lessen existential dread and its influence on depression. TMT research highlights the potential benefits of integrating mortality awareness into mindfulness-based practices [1, 26]. Long-term death recollection practice may foster proactive coping strategies (e.g., mindful problem-solving) rather than avoidant behavior. Supporting this view, prior evidence indicates that long-term meditators exhibit reduced amygdala reactivity in response to stressors—consistent with a moderation analysis pathway [45].

Death Recollection is unfamiliar to many outsides of Thailand, particularly within scientific communities, leading to a scarcity of research on death meditation. The scientific community can learn from Thailand’s rich tradition and practice of death contemplation, although it is important to acknowledge that the broader scientific community has yet to explore or validate these practices [47, 50]. The results of this study show that death recollection moderated the stress-depression relationship, which may contribute to the discussion about death meditation [27] and the benefits of mindfulness meditation on depression that other research has found [13, 17, 39]. The results of this study correspond with the available research about death meditation that found significant reductions in death anxiety following death meditation practice [31]. In a separate study, death anxiety was found to be a strong predictor of psychopathology and depression severity [25]. The mechanism behind death recollection that may result in lower depression is not yet clear, though reductions is death anxiety are likely one reason why. For many in this study’s population, death recollection may have been their first experience placing a timeframe on life, a practice which may have reframed their understanding of life and death.

The recollection of death is a form of cognitive reappraisal that encourages reflection on one’s remaining lifespan. A person’s priorities may change when they consider that the proximity of mortality is near. The importance of everyday stressors and desires is reframed, with potential shifts in emotional responses. Previous research found that under high stress, individuals with greater cognitive reappraisal ability reported fewer depressive symptoms than those with lower ability [48]. A related study found that reflecting on mortality can bring about positive psychological changes, such as prompting contemplation of intrinsic values (that which gives meaning to life and joy) and awakening an appreciation for life’s moments [27]. What sets death recollection apart from other meditation types is the explicit focus on death awareness. According to terror management theory, activated awareness of one’s death prompts a self-preservation instinct of meaning-making to cope with the reality of inevitable death [37]. Positive behavioral change (such as increased mindfulness following death recollection may function as one aspect of this self-preservation instinct. In practice, death recollection also serves as a spiritual practice intended to nurture wisdom about life.

The death recollection meditation is intended within Buddhist philosophy to foster tranquility and wisdom about impermanence [3]. The practice may also support individuals trying to lead lives that refrain from causing harm to themselves and others [51, 53]. Additionally, the practice may nurture the expression of loving-kindness and generosity toward others. It does so by shifting a person’s perspective, fostering gratitude, promoting unity, motivating meaningful actions, encouraging emotional growth, and emphasizing the realization that life is transient and can cease at any moment [47].

Some researchers have acknowledged that vulnerable populations exposed to death awareness could experience heightened anxiety [26, 27], which suggests sensitivity may be necessary for individuals on a case-by-case basis. Death may be a topic some individuals do not wish to contemplate. Though individual preferences vary, university students in one study most frequently rated their orientations toward death as a neutral acceptance (death as a reality to not be feared or welcomed), whereas death avoidance was least often rated (unwillingness to think or talk about it) [44]. Broadly, it may be safe to consider death, with a study on death awareness having found no endocrine stress response to death reminders, indicating the fight or flight sympathetic nervous system was not threatened when death reminders were introduced [4]. As an entry-level step to death recollection, contemplating one’s own death can begin in timeframes starting many years away [27].

The mindfulness component of the death recollection practice may influence psychological changes such as increased mindfulness, self-compassion, and coping ability [18, 32], with stress levels and life satisfaction positively influenced during the COVID-19 pandemic [19]. As a person practices mindfulness and emotional reactions and judgments are accepted and laid aside, a positive feedback loop [1] may form that enhances one’s overall well-being. When more mindful, a person may more flexibly choose better methods to cope with stress [18]. These positive cognitions are important in light of the reciprocal cognitive and biological influences that contribute to the development of depression [21]. Literature about the benefits of higher mindfulness determined that mindfulness moderated the effects of perceived stress on depression [22]. Mindfulness meditation interventions were also found to reduce depression in adolescents [11, 39] and emerging adults [38] compared with controls. Mindfulness, then, may be an important component of death recollection that may help adolescent mental health.

It is the case for most of this study’s participants that meditation acts as a religious practice. Religious practices have been shown to buffer against mental illness during the COVID-19 pandemic [8, 14]. Religious practices were themselves found to be associated with adolescent self-esteem, provided the rituals or activities were relaxed and not too strictly regulated [35]. There may be unknown cognitive learning processes, such as about Buddhist interdependence and about selfhood, often referred to as wisdom among practitioners [12, 56], which may have a positive impact on mental health.

In summary, these findings underscore the complex psychological processes that may influence mental health outcomes, such as about cognitive reappraisal, cultural practices such as death recollection, and broader contextual factors. Together, these insights offer a foundation for future research about the potential applications of death recollection practice.

### Clinical implications

Practically, death recollection may be advisable for people with mild symptoms of depression, potentially for those with a fear of death. Death education has had notable positive effects on cancer patients in palliative care in reducing anxiety, depression, attitudes toward death, and overall quality of life [59], suggesting death recollection may also be safe and useful with vulnerable populations. Combining meditation with death recollection could potentially enhance its effectiveness. Meditation itself has proven to be a protective factor against psychological trauma [58], particularly in contexts such as the COVID-19 pandemic or regional conflicts that may escalate into large-scale crises with significant casualties. Death recollection implementation should be skillfully executed and intertwined with regular meditation practice, and it can be practiced in both clinical and general settings.

### Limitation and future research

This study primarily relied on self-reported data, which may carry inherent biases due to individual perceptions or subjective interpretations.

In addition, due to the nonrandomized design, the authors cannot provide absolute certainty regarding the results. Therefore, it is appropriate to be cautious when interpreting our findings, acknowledging the limitations despite their consistency. Death recollection is a form of mindfulness meditation [3, 46]; therefore, further study of this meditation’s benefits may help us understand how mindfulness influences the experience of death recollection.

Regarding sampling, two Buddhist boarding schools were purposively selected to ensure a sufficient number of meditators. The Buddhist environment, along with encouragement for meditation practice, limits the generalizability of this study’s results to nonreligious environments. Additionally, a larger proportion of females participated, limiting the generalizability of the results by gender to national or regional populations. Advocating for experimental studies with a randomized population to limit bias and explore the impact of death recollection as a preventive factor in diverse settings, such as among older individuals or those predisposed to mental illness, is crucial. Though the analysis controlled for some sociodemographic influences and meditative experiences, other confounding factors such as personality traits, pre-existing mental health conditions, recent life stressors, the participant’s mindfulness levels, and the type of death recollection instruction, may also have influenced this study’s findings. Investigating the efficacy of death recollection in these populations can offer insights into its potential as a preventive measure against mental health challenges. These studies could help identify whether regular engagement in death recollection contributes to resilience or acts as a protective factor against the development or exacerbation of mental health conditions. Conducting experimental research in varied contexts, cultures, and populations will significantly contribute to expanding our understanding of the potential benefits and applications of death recollection as a preventive strategy in mental health.

## Conclusions

The study suggests that death recollection may have an impact on the relationship between stress and depression symptoms among Thai teenagers in boarding schools. These findings connect the lessor known death recollection with possible mental health benefits. Incorporating death recollection into meditation is considered advanced practice in Thailand, though it is accessible to people of all ages. Due to the purposive sampling method, generalizations should be made cautiously. Conducting a replication study is encouraged. In addition, a longitudinal study is recommended to understand death recollection temporal dynamics and potential causal mechanisms related to stress influenced depression symptoms. This would clarify whether long-term practice strengthens moderation over time. The hypothesis is that practitioners who increase death recollection over time will show a progressively weaker stress-depression relationship compared to non-practitioners.

## Data Availability

The datasets used and/or analyzed during the current study are available from the corresponding author upon reasonable request.

## Abbreviations

iSBI: Inner strength-based inventory
OI: Outcome inventory
PSS: Perceived stress scale
